# From Computation to the First-Person: Auditory-Verbal Hallucinations and Delusions of Thought Interference in Schizophrenia-Spectrum Psychoses

**DOI:** 10.1093/schbul/sby073

**Published:** 2019-02-01

**Authors:** Clara S Humpston, Rick A Adams, David Benrimoh, Matthew R Broome, Philip R Corlett, Philip Gerrans, Guillermo Horga, Thomas Parr, Elizabeth Pienkos, Albert R Powers, Andrea Raballo, Cherise Rosen, David E J Linden

**Affiliations:** 1 Department of Psychological Medicine, Institute of Psychiatry, Psychology & Neuroscience, King’s College London, London, United Kingdom; 2 School of Psychology, Cardiff University, Cardiff, United Kingdom; 3 Division of Psychiatry, University College London, London, United Kingdom; 4 Wellcome Trust Centre for Neuroimaging, University College London, London, United Kingdom; 5 Department of Psychiatry, McGill University, Montreal, Quebec, Canada; 6 Institute for Mental Health, School of Psychology, College of Life and Environmental Sciences, University of Birmingham, Birmingham, United Kingdom; 7 Department of Psychiatry,Medical Sciences Division, University of Oxford, Oxford, United Kingdom; 8 Faculty of Philosophy, Humanities Division, University of Oxford, Oxford, United Kingdom; 9 Yale School of Medicine, Yale University, New Haven, CT; 10 Department of Philosophy, The University of Adelaide, Adelaide, South Australia, Australia; 11 Department of Psychiatry, Columbia University, New York, NY; 12 Graduate Institute of ProfessionalPsychology, University of Hartford, West Hartford, CT; 13 Department of Psychology, Faculty of Social and Educational Sciences, Norwegian University of Science and Technology, Trondheim, Norway; 14 Department of Medicine, Division of Psychiatry, Clinical Psychology and Rehabilitation, University of Perugia, Perugia, Italy; 15 Department of Psychiatry, University of Illinois at Chicago, Chicago, IL; 16 Division of Psychological Medicine and Clinical Neuroscience, MRC Centre for Neuropsychiatric Genetics and Genomics, School of Medicine, Cardiff University, Cardiff, United Kingdom

**Keywords:** computational psychiatry, phenomenology, modeling, agency, ownership

## Abstract

Schizophrenia-spectrum psychoses are highly complex and heterogeneous disorders that necessitate multiple lines of scientific inquiry and levels of explanation. In recent years, both computational and phenomenological approaches to the understanding of mental illness have received much interest, and significant progress has been made in both fields. However, there has been relatively little progress bridging investigations in these seemingly disparate fields. In this conceptual review and collaborative project from the 4th Meeting of the International Consortium on Hallucination Research, we aim to facilitate the beginning of such dialogue between fields and put forward the argument that computational psychiatry and phenomenology can in fact inform each other, rather than being viewed as isolated or even incompatible approaches. We begin with an overview of phenomenological observations on the interrelationships between auditory-verbal hallucinations (AVH) and delusional thoughts in general, before moving on to review several theoretical frameworks and empirical findings in the computational modeling of AVH. We then relate the computational models to the phenomenological accounts, with a special focus on AVH and delusions that involve the senses of agency and ownership of thought (delusions of thought interference). Finally, we offer some tentative directions for future research, emphasizing the importance of a mutual understanding between separate lines of inquiry.

*I think everyone hears a voice that’s their thoughts.It’s only when those voices start having their own mind and willpower that you’re hearing voices in your head.*

(paranoid_cataclysm, July 31, 2009; Raballo^15^)

## Introduction

Psychosis is a syndrome characterized by severe distortions in one’s sense of reality. The most prominent symptoms of psychosis are delusions and hallucinations, which are usually defined as fixed false beliefs and perceptions without corresponding external stimuli, respectively. However, such definitions have been critiqued as rather arbitrary if not oversimplistic and as failing to capture the full complexity and heterogeneity of the experiences of psychosis.

Both phenomenological and neuroscientific approaches have attempted, albeit in very different ways, to address some of this complexity in psychotic experiences. However, there is a substantial explanatory gap between these approaches.^1^ One possibility for addressing this gap is computational psychiatry, a newly emerging field that uses formal mathematical models to delineate mechanisms of brain function and disease states.^2^ This approach has the advantage of being able to bridge basic and clinical neuroscience research and potentially offer unifying accounts not only within a given psychiatric disorder but across different symptoms and diagnostic domains.^3^ Here we begin by reviewing the relationships between auditory-verbal hallucinations (AVH) and delusional thoughts in schizophrenia-spectrum psychoses, before offering critical analyses of current theories from different levels of explanation.

We argue that delusional thoughts—in particular, delusions about the agency and ownership of thought (ie, delusions of thought interference)—and AVH are not best viewed as isolated mental events but are intricately related phenomena not only in degree but also in kind, ie, under certain circumstances delusions and hallucinations may not be clearly separable. This view was actually supported by French psychiatrist Esquirol,^4^ who formally introduced the concept of hallucination in psychiatry in the 19th century and whose legacy has sadly been largely forgotten. Esquirol’s notion was that hallucinations are a form of delirium and not a perception that “makes patients *believe* they have a perception (added italics),”^5^ therefore stressing the belief-like and cognitive/intellectual aspects of hallucination. With Esquirol’s original insight in mind, we aim to review computational models of AVH and evaluate how these models are best applied to phenomenological reports of AVH and delusions of thought interference (eg, delusions resulting from an experience of thought insertion), before arguing that these symptoms may just be different manifestations of intrinsically similar neural processes, eg, processes using Bayesian probabilistic inference.^6^ For more details about the background, see Supplementary Materials.

## The Blurred Lines Between Thought and Perception

Although they are usually allocated into separate descriptive silos (ie, according to the conventional dichotomy between aberrant *perception* and *cognition*, respectively), AVH and delusional thoughts share important phenomenological features, such as an autocentric, self-referential architecture and profound alterations of lived space, time, and intersubjectivity.^7–12^ This is particularly manifest in prototypical “voices” (eg, commenting and imperative voices) and transitivistic delusions or thought interference (eg, delusions of control, thought insertion, thought withdrawal, and thought broadcasting), and also in delusions of reference and persecution. Recent empirical analyses^13^ indicate indeed that AVH articulate themselves in an experiential realm that is in-between the phenomenology of cognition and perception, retaining features of both an altered stream of thought and quasi-material aspects of sensorial givenness.^12,14–16,61^

The psychopathological interconnectedness of delusional thoughts and AVH may be somewhat circular (at least in the sense of potential co-perpetuation): the former may increase the proclivity to thematize anomalies of the stream of consciousness as AVH, and, conversely, AVH could promote the further articulation of delusional themes. Moreover, any account that posits delusional thoughts and AVH as descriptively separable symptoms needs to take heed of autobiographic accounts^18^ as well as phenomenological research,^19,20^ suggesting that both are consequences of a psychotic transformation of the medium of consciousness. This was relatively clear for major authors of the last century; eg, Bleuler^21^ emphasized that AVH operate through a comprehensive transitivistic delusional ideation: “the voices not only speak to the patient, but they pass electricity through his body, beat him, paralyze him, take his thoughts away” (1911/50, p.94). Minkowski^22^ characterizes schizophrenia as arising from the *trouble générateur* and a “loss of vital contact with reality.” Even Jaspers,^23^ who suggested primary schizophrenic delusions may be outside the realm of “understandability” (although secondary delusions may still be amenable to empathic understanding), insisted that the task of psychopathology was to focus on “actual conscious psychic events,” rather than isolated, clinician-identified symptoms no matter how un-understandable they may seem to be. Yet, contemporary empirical research has rediscovered such intersection between AVH and delusional thoughts only recently.^13,24^

One prominent contemporary theory of schizophrenia that incorporates this perspective was developed by Sass and colleagues,^25,26^ who view symptoms of schizophrenia as manifestations of a disturbance of *ipseity* or basic self-experience, ie, of being a “vital and self-coinciding *subject* of experience” (p. 428). Thought, perception, and action come to feel strange, awkward, foreign, extrinsic to, and alienated from oneself.^27,28^ Fuchs^29^ has put forward a somewhat different hypothesis, arguing that both AVH and delusional thoughts arise from a transformation of the world in which worldly objects lose their independent existence (ie, they are no longer available to others and perceivable from a variety of other perspectives), which can result in objects seeming to exist only for oneself, as well as the experience of special self-directed meanings or messages associated with objects and events (delusions). In this context, perception-like phenomena that are not in fact accessible to others (hallucinations) may take on an ontological status similar to what others may consider to be “objective” perceptions. (However, as Ratcliffe^30^ notes, these accounts do not address some potentially crucial aspects of hallucinations, including their specific (and often quite negative) content, the perceptual (or quasi-perceptual) qualities of hallucinations (compared to other manifestations of disturbed ipseity), and, perhaps most importantly, the fact that hallucinations occur not only for persons with ipseity disorders (ie, schizophrenia), but also for persons with trauma histories, mood disorders, or no clinical history at all.) It should be noted that hallucinations, at least when described in schizophrenic psychoses, often retain centrality of the self or a kind of solipsistic and subjectivized quality^31–34^ and, for many, are distinguishable from typical perceptions.^35,36^

Indeed, it has been argued that how patients with schizophrenia access conscious information (including an elevated threshold to consciousness) plays a crucial role in the merging of cognition and perception, and it is likely that a unifying mechanism underpins both processes. The proposal of Northoff and Huang^37^ of a temporospatial theory of consciousness (TTC) links the 4 dimensions of consciousness (level/state, content/form, phenomenology/experience, and cognitive processing/reporting) and offers an account of the brain as situated in time and space while constructing its own temporospatial structure. In particular, the concept of “temporospatial *alignment*” is considered disrupted in schizophrenia relating to the content and form of consciousness.^38^ Although the TTC does not explicitly incorporate the relationships between consciousness and self, the brain regions (including cortical midline structures) thought to underlie self-experience are also at least partly responsible for the alignment and integration of conscious experience. This offers tentative evidence that there may be a common basis underlying thought and perception that are embedded in the very structure of consciousness. As Henriksen et al^27^ note, “From a phenomenological perspective, AVHs in schizophrenia are not primarily sensory-perceptual but rather cognitive phenomena arising from a partial dissolution of certain structures of self-consciousness”(p.166).

Such work may be used to frame and guide empirical research on schizophrenia and hallucinations; thus, computational models of hallucinations and other psychotic symptoms should be able to account for and explain not only isolated symptoms but also symptoms as they are experienced, as interrelated and embedded within an overall context of patients’ selfhood and relationship with the world. As Larøi et al^39^ note, future work on hallucinations must acknowledge their status as “meaningfully interrelated facets of a more comprehensive and characteristic gestalt change in the patient’s experience (field of consciousness) and existence” (p. 235). In the section “Modeling AVH: Computational and Cognitive Frameworks,”we review some of the most prominent models in the computational psychiatry of AVH research and consider how they may fit in the wider framework of altered subjective experiences.

## Modeling AVH: Computational and Cognitive Frameworks

Any model of hallucinations must be constrained by empirical neurobiological findings, such as those reviewed in Supplementary Materials. In this section, we first provide a brief overview of a network model of AVH, followed by one of the most dominant cognitive models of AVH (the inner speech model) while integrating it with neuroscientific and computational theories. Finally, we focus on a relatively recent hierarchical Bayesian model of AVH and how it relates to our understanding of AVH.

### Attractor/Network Models

An attractor is a set of configurations to which the states of a dynamical system are drawn.^40^ Examples in neuroscience include the attractors used to model working memory processes in the prefrontal cortex^17^,^41–43^ and oculomotor control^44^ and to account for the activities of hippocampal cells involved in navigation.^45,46^ They have also been used to model features of schizophrenia.^47,48^

The dynamics of a system can be described in terms of its “energy” landscape. Attractors manifest as regions of low energy (basins) in the energy landscape. In a neural network, the shape of the landscape is determined by the strength of connectivity between neuronal populations. This concept complements many other modeling approaches. For instance, theories founded on Bayesian inference either implicitly or explicitly rely on the existence of minima in an energy functional, specifically, a free energy (cf predictive coding^49^) or an approximation of this (cf loopy belief propagation^50^).

Perceptual inference can be formulated as a process of descent from high to low energy.^51–53^ False perceptions, such as hallucinations, might in theory result from changes in the shape of the energy landscape. It has been proposed that disruptions in GABA and NMDA receptor conductance (modulated by dopamine^54^) in the prefrontal cortex could result in such changes.^55^ For example, a reduction of excitatory pyramidal-inhibitory interneuron connectivity would mean that other states are less suppressed when one group of neurons is active. An increase in stochastic fluctuations in firing rates would make it easier to jump from one basin to another.

This proposition potentially accounts for several phenomena in schizophrenia including hallucinations, while also accommodating prominent neurobiological theories of disrupted excitation-inhibition (E-I) balance^56,57^and aberrant dopamine signaling.^58^ An alternative to modulating synapses to disrupt the energy landscape is to remove connections between neurons. Such “pruning” approaches have successfully reproduced (single word) hallucinations in silico, but neither model has so far demonstrated the spontaneous production of more fluent speech.^59^

### Inner Speech/Comparator Model

There are good reasons to think that AVH involve motor processes. Perhaps the most convincing is that they involve the consequences of an action (ie, speech), in contrast to other forms of hallucinations. This is another reason for our focus on AVH, as nonverbal hallucinations are very likely to have different mechanisms.^60,61^ The “comparator” or “inner speech” model is an influential model of AVH based on predictive motor processes—efference copy and the forward model. It proposes that efference copies of motor commands are sent to a “forward model” that uses them to predict their sensory consequences in advance of sensory feedback. Successful prediction may attenuate the perception of those sensory consequences and also result in the feeling of agency for a movement, whereas prediction errors may lead one to infer the environment or some other agent was responsible for the discrepancy. Patients with schizophrenia are known to have problems with prediction (eg, of the motion of targets during smooth pursuit),^62^ and if these problems extend to predicting the consequences of one’s own movements, then the consequent loss of the feeling of agency for self-generated movements could lead to the belief that another agent is responsible for them: ie, passivity symptoms.^63^ In terms of AVH, the sensation that AVH come from someone else/another source other than one’s self could also be accounted for by failures in self-monitoring. The externality and alien nature of AVH are indeed important phenomenological features in many—but not all—cases. This model is bolstered by numerous empirical findings.^64,65^ In addition, the self- and source-monitoring models of AVH have benefited from studies using signal detection theories and emotional processing, where a sense of perceptual hypervigilance linked to heightened emotional states (threat, fear, and anxiety) results in an urgent need to reduce uncertainty of the signal and lowers the threshold of auditory perception, especially when it comes to internally generated stimuli.^66^ Interestingly, this kind of “jumping-to-conclusions” or rapid judgments based on limited sensory or cognitive evidence are also key to delusion formation.^67^

The ipseity disturbance model suggests that such source-monitoring disturbances could be a reflection of the hyperreflexivity or tendency to take tacit acts of consciousness as an object of reflection rather than the implicit medium of awareness of the world^25^ (although Henriksen et al^27^ contest the assumption in source-monitoring theories that patients perceive their hallucinations as real). This model points to a notion of selfhood that it maintains a world- or object-directed intentionality; when intentionality is no longer prereflectively inhabited, thoughts or other intentional acts may become distorted and take on physical or perceptual qualities. Indeed, early experiences of subtle changes in the experience of cognition and stream of consciousness, including difficulties distinguishing between thought and perception, may predict the later development of hallucinations.^68^ Other models suggest that AVH may be more related to a disruption of anticipation of certain kinds of anxiety-producing thoughts: certain unwanted thoughts may be anxiously anticipated and therefore imbued with unusual object- and perception-like qualities.^69^ This model may help to explain the content-specific nature of many AVH, suggesting that it is not all thoughts that feel alienated and external, but only those with particularly disturbing or distressing content. An alternative model may be a better explanation for false inference about communications from another agent; thus, we next discuss the hierarchical Bayesian model of AVH. For some of the critiques on the comparator model, see Supplementary Materials.

### A Hierarchical Bayesian Model of AVH

A popular view of the brain among contemporary neuroscientists is that it instantiates a hierarchical Bayesian model of its environment. This view has several important implications for hallucination research.^70^ First, different levels in the brain’s hierarchical organization represent the causes of sensory data at levels below in an increasingly abstract way as one ascends the hierarchy.^71^ Second, the brain uses or approximates Bayesian inference to infer these causes: meaning it must combine prior beliefs about these causes with sensory data (in the form of a “likelihood”)—weighted by their relative certainty (or “precision”)—to make its inferences, or “posterior beliefs.” Crucially, the incorrect assignment of precision leads to failures of inference, eg, if sensory precision is underestimated or prior precision overestimated, then the prior will dominate the posterior: a potential cause of hallucinations of any sort.^49^ Third, most priors within the model—eg, the expected sound of someone’s voice or the content of their speech—are learned from previous inferences: known as “empirical priors.”

Numerous neural message–passing schemes can perform Bayesian inference. One such is predictive coding, in which descending messages from higher levels are predictions of quantities at lower levels, and ascending messages are prediction errors—the difference between the predicted and the actual values—weighted by their precision.^49^ An alternative is belief propagation, in which the descending and ascending messages are priors and likelihoods.^72^ If the brain uses predictive coding, how might it encode precision? Given precision changes the weights of priors and likelihoods in inference without altering their means, its neural implementation ought to increase the “gain” of neural messages without generating new messages itself. One obvious candidate mechanism is synaptic gain, ie, the factor by which presynaptic input is multiplied to generate postsynaptic potentials. Synaptic gain can influence the precision of encoded states at different scales: both at the neural level, via neuromodulatory receptors such as the *N*-methyl-d-aspartate (NMDA) receptor and receptors for dopamine and acetylcholine, and at the network level, by altering the robustness of neural representations to other inputs or stochastic fluctuations in neural activity. In particular, NMDA receptors on inhibitory interneurons—thought to be dysfunctional in schizophrenia^73^—may determine the extent to which one neural “explanation” for sensory input can suppress competing “explanations.”

Many paradigms—eg, visual illusions, oculomotor pursuit tasks, EEG oddball tasks, sensory attenuation tasks, and belief updating tasks—indicate that patients with schizophrenia have an imbalance between the precision of priors (too low) and sensory evidence (too high).^74^ In other paradigms in schizophrenia, however, priors dominate sensory evidence^75,76^—interestingly, in both cases these were recently learned perceptual priors. There are various possible explanations for these apparently opposite imbalances in different paradigms. One is that the hierarchy is so deep that, eg, decreases in midlevel synaptic gain could be expressed as decreased precisions of either priors (for the levels below, which receive predictions from this level) or likelihoods (for the levels above, which receive prediction errors from this level), depending on whether the paradigm depends more on lower or higher levels for its effects. Another is that schizophrenia may involve a failure of adaptive *control* of synaptic gain, rather than simply too much or too little at different levels. Belief propagation account of Jardri and colleagues proposes these imbalances come about due to priors or likelihoods being “overcounted” due to disinhibited message passing,^76–78^ rather than alterations in synaptic gain, although this and other belief propagation models also contain precision terms that could be encoded by synaptic gain.

Under a hierarchical Bayesian account of AVH, hallucinations must result from overprecise priors that are not corrected by (relatively imprecise) likelihoods (ie, the precision of the mapping between auditory input and its possible causes is low).^71^ This is because a false-positive inference requires that a percept is internally generated and is not derived from ascending sensory information. An interesting question is where such priors might be: Are they at the higher levels of the model (eg, prefrontal cortex), imposing themselves on middle levels, or are they at middle levels (eg, superior temporal cortex), imposing themselves on lower levels (ie, empirical priors)? The latter could be an instance of failure to attenuate sensory precision (ie, synaptic gain), as is found in the force-matching paradigm.^64,65,79^ In this case, increased precision/gain may be of autonomous neural activity in higher auditory cortex (as proposed by “attractor state” models reviewed in “Attractor/Network Models” section) rather than of sensory input. The former case may result from a compensatory increase in prior precision.^80^ Recent modeling of the former option using a Bayesian framework indicates that AVH can occur when an agent engaging in dialogue has a strong prior belief that it is listening to a voice and an imprecise likelihood mapping. When it expects to hear a voice but none is present, it generates a hallucinated voice to satisfy its expectations.^81^ The fact that this expected voice is that of a conversational partner may also play a role in explaining aberrancies of agency in AVH.^81^

Numerous kinds of priors could contribute to AVH. A large cluster analysis of AVH phenomenology (largely schizophrenia) indicates different subtypes exist—memories, first-person AVH, other-person AVH, and unintelligible sounds^82^—even within one person. This suggests that the common pathology lies in an area that receives predictions about speech from multiple sources (ie, superior temporal cortex) but is unable to sufficiently attenuate their precision, such that these priors dominate resulting percepts. This will especially be the case if the precision of incoming sensory data is low. In the case of memories and first-person AVH, predictions are likely to come from medial temporal lobe and a network of frontal areas including inferior frontal gyrus^40,83^ (also associated with “inner speech”), respectively.

Given that AVH develop increasing complexity over time,^84^ the likeliest explanation is that identities of other-person voices are empirical priors that are inferred from previous hallucinations. For example, hearing a muffled insult might create an image of someone whose voice that might be, which may generate expectations about other insults. The development of empirical priors concerning the origins of AVH will have a critical impact on the extent to which the hallucinating subject feels he or she has control over the AVH—a key distinguishing factor between psychotic and healthy voice-hearers.^84,85^ If voices that could be ascribed to distorted perceptions or hallucinations are instead attributed to an external agent, the voice-hearer’s sense of autonomy will diminish.

Another key phenomenological difference is the emotionally negative content (usually) associated with clinical or psychotic voice-hearers; although this has not yet been systematically studied under the Bayesian framework, there is accumulating evidence pointing toward the roles played by past memories (eg, of trauma), which may act as top-down expectations relating to voice content,^80,86^ especially in memory-related subtypes of AVH.

**Fig. 1.**
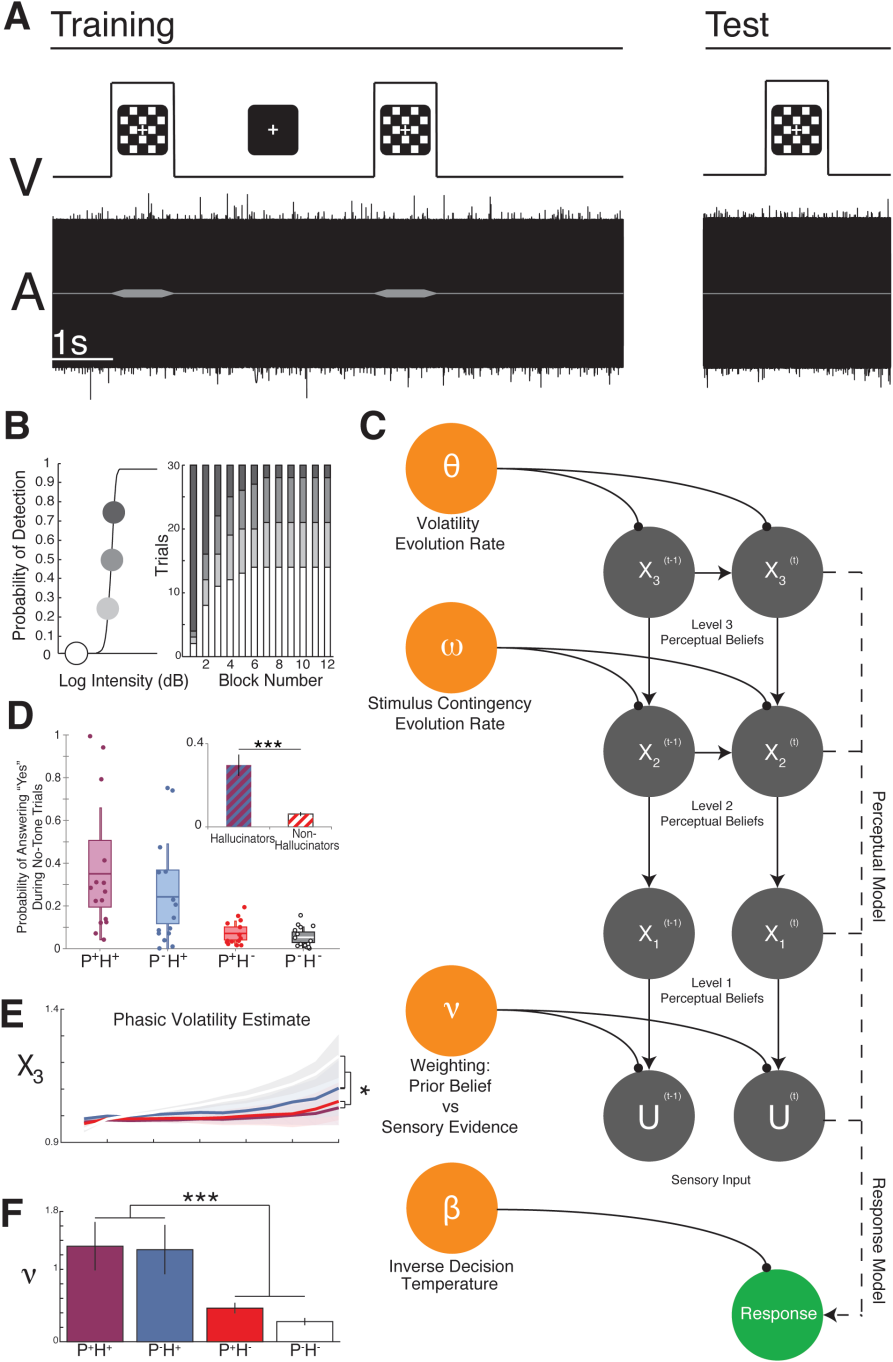
A hierarchical Bayesian model of conditioned hallucinations. Reproduced with permission.In this study,^88^ subjects with and without psychosis (P^+^, P^−^, respectively) and with and without auditory verbal hallucinations (H^+^, H^−^, respectively) were conditioned to associate tones presented at threshold with a concurrent checkerboard stimulus (A). This association was then tested by presenting the checkerboard alone and recording subjects’ reports of hearing the tone: the probability of presentation of a subthreshold or absent tone increased over blocks (B). Subjects with hallucinations (irrespective of psychosis) were more likely to hallucinate tones (D, ***P<.001). The evolution of this association over time was modeled using a hierarchical Bayesian model (C), in which the first level (X_1_) is the belief in the presence of the tone, the second level (X_2_) is the association of the tone with the checkerboard, and the third (X_3_) is the rate of change of the association, and subject-specific parameters ω and θ affect how quickly these beliefs change at levels 2 and 3. At X_3_, there was a significant block-by-psychosis interaction (E, *p< 0.05). The belief about having heard a tone (ie, at X_1_) on one trial—in Bayesian terms, the “posterior”—then becomes the prior belief in the next trial, and the weighting of this prior belief over the incoming sensory evidence is parameterized by ν. Crucially, ν was higher in hallucinators (irrespective of psychosis): ie, hallucinators overweighted their (empirically learned) prior beliefs in this task (F, ***P<.001). One could likewise model voice perception using a hierarchical model in which inputs are sounds and higher levels encode beliefs about phonemes, words, sentences, and speaker identities. In such a model, verbal hallucinations could result from a similar overweighting of prior beliefs.^104^

This model makes predictions that cohere with empirical investigations: (1)Loss of NMDA receptor function on inhibitory interneurons in temporal areas might lead to an increase in resting activity (it is harder to suppress “noise”) but a decrease in task-related (including prediction error-related^86^) activity (task-related activity is inversely correlated with the baseline level)—both of which have been demonstrated in functional magnetic resonance imaging studies with schizophrenia patients with AVH,^86^ but are hard to explain using the “comparator” model.^80^(2)Inhibitory interneuron dysfunction in schizophrenia may make it harder for auditory cortex to generate γ oscillations in response to 40-Hz tones (which depend on interactions between pyramidal cells and interneurons).^87^ (3)Voice-hearers ought to demonstrate more precise empirical priors than controls—this has been shown in both hallucinating patients with schizophrenia and healthy voice-hearers, using tasks in which learned contexts influence uncertain sensory data^88^ (see figure 1; also Cassidy et al^89^).

The Bayesian model of AVH may have phenomenological correlates in the notion of aberrant salience, which reflects a tendency to attend to irrelevant or background perceptual details (as opposed to details that are more relevant for goal-oriented behavior). It has been suggested that persons with schizophrenia may demonstrate this anomalous processing of perceptual stimuli. Such attentional and perceptual disturbances may result in the assignment of greater importance to irrelevant stimuli,^90^ rather than allowing one’s attention to be directed (and corrected) by more accurate information and prior knowledge about the environment. The ipseity disturbance model suggests that this salience disruption results from a loss of “grip” or “hold” on one’s engagement with the environment, whereby disturbances in basic selfhood are likely to disrupt one’s pragmatic and goal-oriented engagement with the world.^26^ It may also impact the inability to appropriately correct or update one’s interpretations about various perceptual inputs, and the tendency to interpret ambiguous or “noisy” stimuli according to strong overprecise (perhaps emotionally salient) priors.^69^

## AVH and Delusions of Thought Interference

Whether a thought has vocal, audible, or agentive qualities may partly account for the content of the delusion and the way it is reported in research protocols and clinical encounters. Typically, audible voices will be described as AVH and soundless voices as thought insertion.^12^ Yet such descriptions may impose an artificially precise structure on an intrinsically ambiguous form of experience, which also includes emotional distress and the elaborations, interpretations, and defenses produced by the patient. The above theories are compelling in their ability to explain the development and maintenance of AVH from the perspectives of neurobiology, perceptual and motor systems, and cognitive modeling. In this section, we consider how some of these models might be able to shed light on several major phenomenological features of AVH and thought insertion, especially findings of their interrelationship and experiential similarities.

In thought insertion, the subject reports that thoughts arrive in their mind *“*out of nowhere.”^91^ Most explanations of thought insertion descend from source-monitoring accounts of intentional action.^92,93^ These accounts distinguish sense of ownership (the experience that the action belongs to oneself, that the bodily movement is one’s own) from sense of agency (the experience of intending and controlling the action).^94,95^ When sense of agency is absent, but sense of ownership is intact, the patients experience their body performing actions, which are nonetheless not felt to be theirs. This way of conceptualizing the experience treats sense or ownership as a form of subjective awareness of bodily occupation of space.^96^ It also is worth noting that some authors^94^ propose the sense of agency and ownership are in fact 2-fold, consisting of a “feeling” and a “judgment”that incorporate bottom-up sensory information (feeling) and top-down beliefs (judgment). From this perspective, one could have a mere feeling of intending the movement but deny the movement is indeed initiated by themselves (due to higher-order factors or conflicts perhaps), such as in experiences associated with passivity phenomena. Here, we use the term “sense” to refer to the prereflective *feeling* of agency/ownership in Synofzik’s account.

Source-monitoring accounts of thought insertion decompose the experience of subjectivity into a sense of ownership and a sense of agency for *thought* (SOT and SOAT, respectively). Thought insertion, on this view, derives from the experience of SOT for thought unaccompanied by SOAT.^97^ There is, however, no straightforward analogy between thoughts and actions. Actions are monitored, at different levels of cognition, for consistency with intentions, generating (in this account) a sense of agency. But we do not, in general, intend our thoughts. So SOAT cannot depend on comparison of actual and intended/predicted thoughts.^98^

Nonetheless, the analogy has been pursued in different ways, each of which argues that a characteristic SOAT goes missing in thought insertion, leaving SOT for thoughts intact. The source of a SOAT might be a sense of agency for speech production (on the plausible assumption that the thoughts in question are episodes of inner speech),^99,100^ protention (online anticipation of the temporal structure of episodes of thought)^101^ or ego-tonia (compatibility with self-representation).^102^ The suggestion is that a monitoring process fails to detect a match between an occurrent thought and the relevant generative process, as outlined in the “Inner Speech/Comparator Model”section.^103^ Another proposal is that source monitoring is between background psychology (understood as the totality of tacit representations on which thinking depends) and conscious thought.^96^

A difficulty with all these proposals is accounting for normal and pathological (eg, in obsessive compulsive disorder) cases of unbidden or intrusive thoughts. Such thoughts arise in the mind in an unpredicted manner but are not experienced or explained as inserted. Another difficulty with such proposals is more fundamental. To preserve the Cartesian intuition, their proponents divide subjectivity into SOAT and SOT, arguing that SOT is intact in thought insertion. It is not clear that this reflects patients’ experience. Patients say that the thoughts are “not theirs.”^91,97^ If we take this seriously, then perhaps the idea of an intact SOT with its connotation of intact “inner space” or mental boundaries needs to be rethought. One possibility, consistent with recent Bayesian approaches to cognition, is that the boundary of inner space is not an intrinsic or given feature of cognition but is actively constructed by the mind. Henriksenet al^27^ suggest that persons diagnosed with schizophrenia experience their interiority as an unusually concrete form of inner space, possibly due to disruption of a natural, embodied relation to exterior space, with the result that thoughts are given spatial, object-like qualities. Similarly, whether a representation is experienced as internal or external to the mind may depend on the predictive model deployed for the task; persons diagnosed with schizophrenia may inappropriately use external models for internal events.

Some ingenious paradigms addressed this issue by defining neural correlates of AVH and thought insertion. Jardri et al^101^ found that transcranial magnetic stimulation over the left temporal–parietal junction (TPJ) reduced AVH and the right TPJ improved agency for inner speech. This result is consistent with others suggesting that sense of agency depends on modulation of inferior parietal areas by TPJ. Note however that the subjects here did not report thought insertion. The clearest result in this area is from an experiment contrasting hypnotically induced thought insertion, which found “specifically, reduced activity in language production regions, and *not* over-activation of cerebellar-parietal regions, was present during thought insertion” (added italics).^100^ The same experiment found reduced activation in supplementary motor area in experience of both thought insertion and delusions of alien control.

Interestingly, phenomenological theories of psychosis provide little clarification regarding similarities and differences between AVH and thought insertion. One suggestion is that there is little or no meaningful difference between these two phenomena, or at least between thought insertion and the more thought-like, inner speech forms of AVH.^69,105^ Instead, it is suggested that the different ways they are represented or described by patients may simply reflect individual or cultural differences in the way anomalous experiences are conceptualized and communicated.^105,106^ However, advances in psychosis research including those on neural computation might be useful for clarifying the shared (or disparate) phenomenology of thought insertion and various forms of AVH.

## Conclusion

Some tentative suggestions for further research and limitations of the current approach can be found in Supplementary Materials. In this conceptual review, we present some of the phenomenological observations and computational models in the study of AVH and delusions of thought interference in psychosis. These have a particular focus on the merging between (delusional) thought and (hallucinatory) perception, which are embedded in current theories of both computational psychiatry and phenomenological psychopathology. We argue that these two approaches are indispensable to advancing the study of psychosis and urge researchers and clinicians to keep the patients’ reality in mind when considering models and explanations. We are fully aware that incorporating different lines of inquiry is only the beginning of a dialogic process between seemingly disparate fields in psychopathology research, and there is no single approach that can account for the sheer complexity of mental illnesses such as schizophrenia. Nevertheless, interdisciplinary research and integrative approaches are crucial in creating a mutual understanding and facilitating collaborative efforts, with the ultimate goal of maximizing benefits to the quality of life of patients with psychosis. The realization that phenomenology and computation work in conjunction and inform each other is undoubtedly a vital first step toward achieving this goal.

## Funding

This work was supported by a Medical Research Council (UK) Doctoral Training Grant (MR/K5013471/1) allocated to C.S.H.; D.E.J.L. is supported by the MRC Centre for Neuropsychiatric Genetics and Genomics (G0800509), Cardiff University; R.A.A. is funded by the Academy of Medical Sciences (AMS-SGCL13-Adams) and the National Institute of Health Research (CL-2013-18-003); T.P. is funded by the Rosetrees Trust (173346); D.B. is supported by the Richard and Edith Strauss Foundation and the Fonds de Recherche du Québec- Santé.

## Supplementary Material

Supplementary Materials
