# Supplementary material for: From Computation to the First-Person: Auditory-Verbal Hallucinations and Delusions of Thought Interference in Schizophrenia-Spectrum Psychoses
Source: Schizophr Bull. 2019 Feb 1;45(Suppl 1):S56–66. doi: 10.1093/schbul/sby073 (PMC6357975; doi:10.1093/schbul/sby073)
Supplement: Supplementary Materials [file sby073_suppl_supplementary_materials.doc]

**From Computation to the First-person: Auditory-Verbal Hallucinations and Delusions of Thought Interference in Schizophrenia-Spectrum Psychoses**

**Supplementary Materials**

***Rationale and Theoretical Premise***

AVH instead of all auditory hallucinations were chosen to be the focus of discussion mainly because their language and speech-like elements fit well with the computational models, and also to avoid an over-ambitious premise beyond what we can offer in a single review. In addition, the frequently self-referential nature of AVH is less relevant or obvious with non-verbal hallucinations but is central to our arguments. Furthermore, although many of the thought interference symptoms in the current review are traditionally classed as Schneiderian first rank symptoms (FRS) and separated from delusional thoughts^1,2^, the emphasis is on the kinds of thought interference that have been given a delusional elaboration or explanation (hence what we call *delusions* of thought interference). On the other hand, some subtypes of AVH (commenting voices, voices heard arguing amongst themselves) are also classed as FRS and closely correlate with overall FRS^3^. It may be tempting to treat all the symptoms mentioned under the umbrella term of FRS, but we did not choose to do so because we considered it necessary, for the clarity of argument, that in the current review the focus is moved away from the concept of FRS.

We are aware that AVH, psychosis and schizophrenic disorders are not synonymous^4^ and therefore have decided to focus on AVH in the context of schizophrenia-spectrum psychotic disorders for better specificity, as expanding further to other disorders will prove far too ambitious to fit within the remit of a single framework. Nevertheless, we believe this collaborative effort will have the potential to unite disparate theories and build a bridge between neuroscience and accounts from phenomenology.

Phenomenological approaches to psychopathology have long recognized the limitations of symptom-focused approaches to psychiatric disorders. In the phenomenological tradition, Husserl refers to the basic sense of shared intersubjective reality as the ‘natural attitude’, a default common-sense attitude. According to this view, we typically believe that things we encounter in our day-to-day world are as they present themselves, are real, and will interact in our common-sense, physical conception of the world^5, 6^. Phenomenological approaches to psychopathology attempt to bracket the natural attitude in order to understand how the world appears to those with various psychiatric conditions. Thus, it tends to focus on the overall transformations of subjectivity that characterise mental disorders, viewing symptoms as facets of experience that “are interdependent in a mutually constitutive and implicative manner” and that arise from an “experiential expressive whole”^7^.

***Neurobiological Considerations***

Structural studies in AVH have consistently found reduced grey matter volume in areas such as the superior temporal gyrus, which contains Wernicke’s area and the primary auditory cortex^8, 9^, as well as in medial and inferior frontal cortex^9^. Reduced grey matter volume in some of these areas has been directly related to symptom severity^8, 10^. Structural covariance between frontal, temporal, hippocampal and insular areas were also related to hallucination severity, strengthening the evidence that a malfunctioning network of language and executive regions is responsible for AVH^11, 12^. Healthy voice hearers - those who hear voices but are not diagnosed with any psychiatric disorder - may show an intermediate degree of grey matter reduction in the superior temporal gyrus^12^.

Alterations in white matter tracts have been consistently demonstrated. In particular, the arcuate fasciculus (AF) has been found to be disrupted in structural^24^ and diffusion studies^12-16^ and, while white matter deficits are present in both hallucinating and non-hallucinating patients, AF disturbances seem specific to patients with AVH^34^, in whom AF integrity is negatively correlated with more severe AVH symptoms^18^. Furthermore, healthy voice hearers have an intermediate phenotype, with reduced AF integrity compared to normal controls but greater compared to patients^19, 20^, suggesting that lowered AF integrity is necessary for hallucinations but that damage to other areas is necessary to produce psychosis^21^. Other AVH-related white matter disturbances have been found in uncinate, thalamic, and callosal tracts^12^.

Functional imaging studies have shown impaired deactivation of the default mode network (a large functional network that usually engages in a state of wakeful rest when there is no active task involved; a so-called ‘task-negative’ network thought to be anticorrelated with ‘task-positive’ networks such as the central executive network) during tasks in patients experiencing psychosis^17^, and increased connectivity between anterior cingulate and superior temporal cortex during self-generated speech^22^, pointing to a network prone to inappropriate activation. The subjective reality of AVH was also related to the functional connectivity between auditory cortex, inferior frontal gyrus (including Broca’s area), the cingulate cortex, the ventral striatum, and other regions^23^. fMRI studies of AVH have also shown fronto-temporal dysconnectivity^22^, as have those using EEG (reviewed in the Predictive Coding section).

Taken together, these results suggest that AVH production requires the dysfunction and dysconnectivity of a distributed network with sensory, motor and attentional components. Dysfunction of some brain regions seems to interact with white-matter-related dysconnectivity to produce AVH phenotypes that differ based on the degree of dysfunction and the specific mix of dysfunctional systems. A model of AVH must be informed by this pattern of functional alterations while accounting for findings in healthy voice hearers.

***Limitations of the Comparator Model***

Despite its success, there are some general problems with the comparator theory of passivity symptoms, and some specific problems with the related model of AVH in schizophrenia. The former stem from criticisms of the comparator account of the sensory attenuation effect^23^. One fundamental issue is that during movement, sensory attenuation also occurs to unexpected stimuli that are *externally* generated and thus cannot be predicted by a forward model^24, 25^ – the N1 is an electrophysiological example of this^26^ – and to stimuli that occur a few hundred milliseconds either side of the movement^27^. Therefore, the attenuation of movement-related sensations cannot occur as a result of them precisely matching predictions (such that any error could be used to ‘tune’ a forward model to make even more accurate predictions in future): it seems a general phenomenon. This does not imply that prediction of action consequences is normal in schizophrenia^28^, but it does mean the loss of sensory attenuation in schizophrenia cannot be (uniquely or even mainly) owed to forward model deficits. If it were, motor control in schizophrenia ought to be much worse than it is, given the size of the effect^29^.

An additional problem for the ‘inner speech’ model of AVH is that there are no ‘reafferent (auditory) speech sensations’ to compare with the ‘predicted speech sensations’, and the notion of ‘reafferent inner speech’ seems redundant^30^ (similar points have been raised about the application of this model to thought insertion^31, 32^). This issue aside, the ‘inner speech’ model seems a possible explanation for hearing one’s own thoughts out loud, or thought echo, but the inference that another agent is saying things whose content doesn’t resemble one’s typical inner speech warrants an additional, or alternative, explanation.

Phenomenologically, a problem with the ‘inner speech’ model is that it cannot fully account for why some AVH are clearly ‘heard’ whereas others are more akin to ‘a sense of being spoken to’ or simply a transmission of silent messages without any sensory component (i.e. soundless voices^33^). Although the latter type of AVH may not qualify as ‘true hallucination’ in its most traditional definition, it nevertheless accounts for a significant amount (over 40%) of the AVH experience^34^. Further, soundless external voices share important similarities with the experience of thought insertion^35^, and both frequently lead to delusional elaborations.

***Directions for Future Research***

These theoretical considerations raise numerous empirical questions, many of which require large scale and detailed phenomenological studies of both schizophrenia and psychosis-like experiences in healthy individuals. Can such studies discriminate between different phenotypes of AVH both within and across groups? If so, do these groups all share reduced sensory attenuation, particularly in the auditory domain? Likewise, do they share electrophysiological signatures of unstable attractor dynamics in superior temporal cortex? Similarly, can such studies provide more raw data on how AVH develop over time, and their relationship with other thought interference symptoms? One avenue might be to study the co-occurrence, treatment response, overlapping/convergent neural mechanisms or even co-heritability of AVH and delusions of thought interference. In this regard, much-neglected phenomenology ought to do for computational psychiatry what epidemiology has done for medicine: provide large scale datasets that permit detailed analysis of associations and hence the generation of causal models of symptoms.

***Limitations***

There are several limitations and potential difficulties associated with this dual approach. First, the models of AVH reviewed in the current article do not make a distinction between conscious and subliminal processing, the latter of which is considered preserved in schizophrenia^36^. In other words, sense of agency as a conscious aspect of motor performance is disrupted even though the automatic or subliminal aspects of motor behavioural remain intact^37^. Second, transdiagnostic features of AVH between schizophrenia-spectrum psychoses and other disorders (e.g. dissociative disorders, borderline personality disorder) are not discussed. Although we set out to be specific about the types of AVH models could account for, this may nevertheless limit the clinical implications of our approach. We are aware it would not be possible to be fully comprehensive or inclusive in any single endeavour; rather than offering a fully formed solution, it is perhaps more important to strive towards the beginning of a dialogue.

**References**

1. Schneider K*. Clinical psychopathology*. Grune & Stratton; 1959
2. Carpenter Jr, W. T., & Strauss, J. S. (1974). Cross-cultural evaluation of Schneider’s first-rank symptoms of schizophrenia: a report from the International Pilot Study of Schizophrenia. *American Journal of Psychiatry*, *131*(6), 682-687.
3. Gur, R. E., Mozley, P. D., Shtasel, D. L., Cannon, T. D., Gallacher, F., Turetsky, B., ... & Gur, R. C. (1994). Clinical subtypes of schizophrenia: differences in brain and CSF volume. *American Journal of Psychiatry*, *151*, 343-343.
4. Waters, F., Blom, J. D., Jardri, R., Hugdahl, K., & Sommer, I. E. C. (2018). Auditory hallucinations, not necessarily a hallmark of psychotic disorder. *Psychological Medicine*, *48*(4), 529-536.
5. Broome MR. *The Maudsley reader in phenomenological psychiatry*. Cambridge, UK: Cambridge University Press; 2013.
6. Broome M. Reality, realness, and the natural attitude. *Philosophy, Psychiatry, & Psychology* 2012;19(2):115-118.
7. Parnas J, Sass LA, Zahavi D. Rediscovering psychopathology: the epistemology and phenomenology of the psychiatric object. *Schizophrenia Bulletin* 2012; 39(2):270-277.
8. Mørch-Johnsen L, Nesvåg R, Jørgensen KN, et al. Auditory Cortex Characteristics in Schizophrenia: Associations With Auditory Hallucinations. *Schizophrenia Bulletin* 2016;43(1):75-83.
9. Kubera KM, Sambataro F, Vasic N, et al. Source-based morphometry of gray matter volume in patients with schizophrenia who have persistent auditory verbal hallucinations. *Progress in Neuro-Psychopharmacology and Biological Psychiatry* 2014;50:102-109.
10. Allen P, Modinos G, Hubl D, et al. Neuroimaging auditory hallucinations in schizophrenia: from neuroanatomy to neurochemistry and beyond. *Schizophrenia Bulletin* 2012;38(4):695-703.
11. Modinos G, Vercammen A, Mechelli A, Knegtering H, McGuire PK, Aleman A. Structural covariance in the hallucinating brain: a voxel-based morphometry study. *Journal of Psychiatry & Neuroscience: JPN* 2009;34(6):465.
12. Bohlken MM, Hugdahl K, Sommer IE. Auditory verbal hallucinations: neuroimaging and treatment. *Psychological Medicine* 2017;47(2):199-208.
13. Geoffroy PA, Houenou J, Duhamel A, et al. The arcuate fasciculus in auditory-verbal hallucinations: a meta-analysis of diffusion-tensor-imaging studies. *Schizophrenia Research* 2014;159(1):234-237.
14. McCarthy-Jones S, Oestreich LK, Bank AS, Whitford TJ. Reduced integrity of the left arcuate fasciculus is specifically associated with auditory verbal hallucinations in schizophrenia. *Schizophrenia Research* 2015;162(1):1-6.
15. Knöchel C, O'Dwyer L, Alves G, et al. Association between white matter fiber integrity and subclinical psychotic symptoms in schizophrenia patients and unaffected relatives. *Schizophrenia Research* 2012;140(1):129-135.
16. de Weijer AD, Mandl RC, Diederen KM, et al. Microstructural alterations of the arcuate fasciculus in schizophrenia patients with frequent auditory verbal hallucinations. *Schizophrenia Research* 2011;130(1):68-77.
17. Gavrilescu M, Rossell S, Stuart GW, et al. Reduced connectivity of the auditory cortex in patients with auditory hallucinations: a resting state functional magnetic resonance imaging study *Psychological Medicine* 2010;40(7):1149-1158.
18. Ćurčić-Blake B, Nanetti L, van der Meer L, et al. Not on speaking terms: hallucinations and structural network disconnectivity in schizophrenia. *Brain Structure and Function* 2015;220(1):407-418.
19. de Weijer AD, Neggers SF, Diederen K, et al. Aberrations in the arcuate fasciculus are associated with auditory verbal hallucinations in psychotic and in non‐psychotic individuals. *Human Brain Mapping* 2013;34(3):626-634.
20. Baumeister D, Sedgwick O, Howes O, Peters E. Auditory verbal hallucinations and continuum models of psychosis: A systematic review of the healthy voice-hearer literature. *Clinical Psychology Review* 2017;51:125-141.
21. Simons JS, Garrison JR, Johnson MK. Brain Mechanisms of Reality Monitoring. *Trends in Cognitive Sciences* 2017; 21(6): 462-473.
22. Mechelli A, Allen P, Amaro E, et al. Misattribution of speech and impaired connectivity in patients with auditory verbal hallucinations. *Human Brain Mapping* 2007;28(11):1213-1222.
23. Brown H, Adams RA, Parees I, Edwards M, Friston K. Active inference, sensory attenuation and illusions. *Cogn Process*. 2013;14(4):411-427.
24. Voss M, Ingram JN, Wolpert DM, Haggard P. Mere expectation to move causes attenuation of sensory signals. *PloS One*. 2008;3(8):e2866.
25. Rushton DN, Rothwell JC, Craggs MD. Gating of somatosensory evoked potentials during different kinds of movement in man. *Brain J Neurol*. 1981;104(3):465-491.
26. Lange K. The reduced N1 to self‐generated tones: An effect of temporal predictability?. *Psychophysiology* 2011;48(8):1088-95.
27. Bays PM, Wolpert DM, Flanagan JR. Perception of the consequences of self-action is temporally tuned and event driven. *Curr Biol CB*. 2005;15(12):1125-1128.
28. Synofzik M, Thier P, Leube DT, Schlotterbeck P, Lindner A. Misattributions of agency in schizophrenia are based on imprecise predictions about the sensory consequences of one’s actions. *Brain J Neurol*. 2010;133(Pt 1):262-271.
29. Frith C. Explaining delusions of control: the comparator model 20 years on. *Conscious Cogn*. 2012;21(1):52-54.
30. Swiney L, Sousa P. A new comparator account of auditory verbal hallucinations: how motor prediction can plausibly contribute to the sense of agency for inner speech. *Front Hum Neurosci*. 2014;8:675.
31. Gallagher S. Neurocognitive models of schizophrenia: a neurophenomenological critique. *Psychopathology*. 2004;37(1):8-19.
32. Sterzer P, Mishara AL, Voss M, Heinz A. Thought Insertion as a Self-Disturbance: An Integration of Predictive Coding and Phenomenological Approaches. *Front Hum Neurosci*. 2016;10:502.
33. Jones, S. R. (2008). Do we need multiple models of auditory verbal hallucinations? Examining the phenomenological fit of cognitive and neurological models. *Schizophrenia Bulletin*, *36*(3), 566-575.
34. Nayani TH, David AS. The auditory hallucination: a phenomenological survey. *Psychol Med*. 1996;26(1):177-189.
35. Billon, A. (2013). Does consciousness entail subjectivity? The puzzle of thought insertion. *Philosophical Psychology*, *26*(2), 291-314.
36. Berkovitch, L., Dehaene, S., & Gaillard, R. (2017). Disruption of conscious access in schizophrenia. *Trends in Cognitive Sciences*, *21*(11), 878-892.
37. Voss, M., Chambon, V., Wenke, D., Kühn, S., & Haggard, P. (2017). In and out of control: brain mechanisms linking fluency of action selection to self-agency in patients with schizophrenia. *Brain*, *140*(8), 2226-2239.
